# Homocysteine—a retrospective and prospective appraisal

**DOI:** 10.3389/fnut.2023.1179807

**Published:** 2023-06-13

**Authors:** Andrew McCaddon, Joshua W. Miller

**Affiliations:** ^1^Faculty of Social and Life Sciences, Wrexham Glyndwr University, Wrexham, United Kingdom; ^2^Department of Nutritional Sciences, School of Environmental and Biological Sciences, Rutgers University, New Brunswick, NJ, United States

**Keywords:** homocysteine, folate, vitamin B12, inborn errors of metabolism, neural tube defects, vascular disease, Alzheimer’s disease, dementia

## Abstract

The biologically important amino acid homocysteine links sulfur, methionine, and one-carbon metabolism. This review describes its initial discovery, the identification of the clinical condition of “homocystinuria” and the recognition of its close relationship to folate and vitamin B12 metabolism. It discusses the history behind its current association with diverse diseases including neural tube defects, cardio- and cerebrovascular disease and, more recently, dementia and Alzheimer’s Disease. It also explores current controversies and considers potential future research directions. It is intended to give a general overview of homocysteine in relation to health and disease.

## Introduction

**Figure 1:**
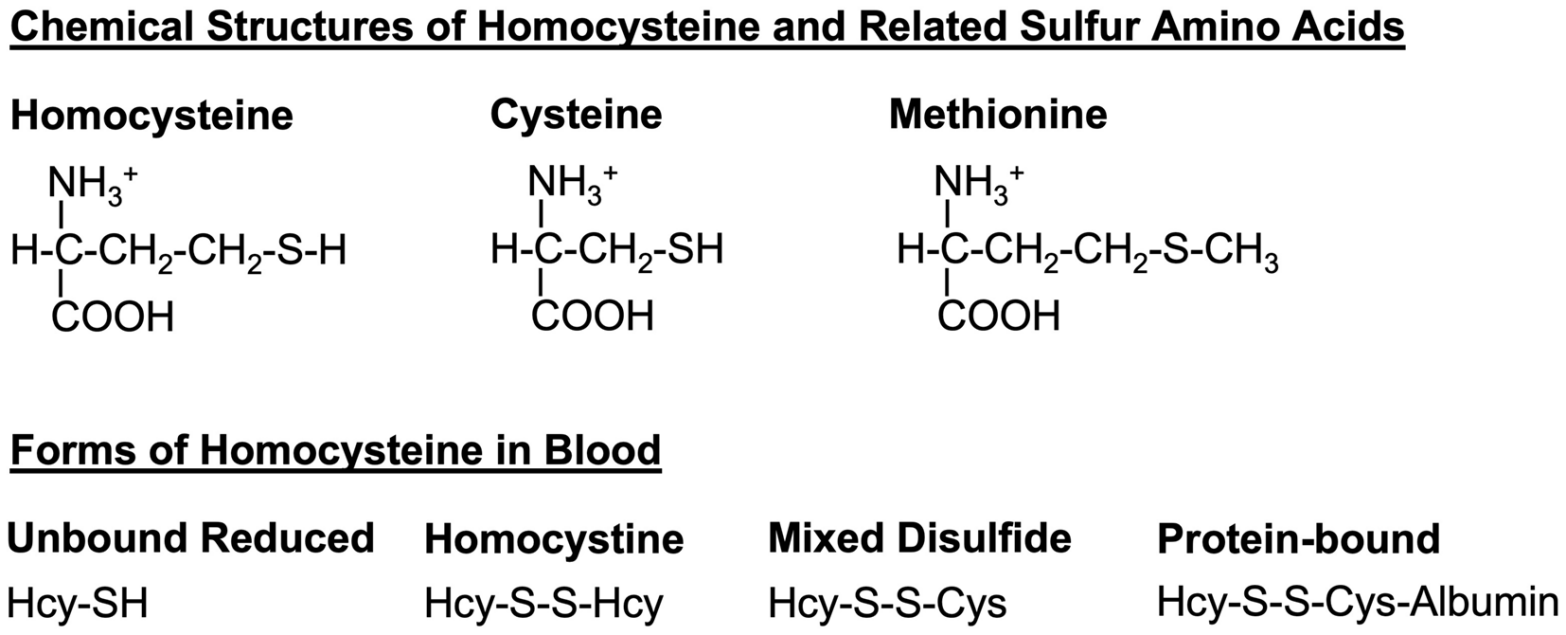
Homocysteine and related sulfur amino acids. Homocysteine, cysteine, and methionine are related amino acids that all contain a sulfur atom in their sidechains. Dietary methionine is converted to homocysteine and to cysteine via the transsulfuration pathway (see Figure 2). In the blood, homocysteine exists in several forms, including the free reduced form (no disulfide bond), homocystine (disulfide bond between two homocysteine molecules), mixed-disulfide (disulfide bond between homocysteine and cysteine), and protein-bound (disulfide bond between homocysteine and a cysteine within a protein such as albumin). Hcy, homocysteine; Cys, cysteine.

The amino acid homocysteine [Figure 1; (1)] was first identified 90 years ago by Butz and du Vigneaud during their investigation of decomposition products of methionine (2, 3). It is now recognized as a biologically important amino acid linking sulfur, methionine, and one-carbon metabolism. Since its identification, over 28,000 research papers have described its relevance to several inborn errors of metabolism, B vitamin status, and diseases as diverse as cardio- and cerebrovascular disease, dementia, renal disease, thyroid disease, and pregnancy complications, to name but a few.

Important developments in the field over this time include the first clinical descriptions of the inborn error of metabolism disorder “homocystinuria” and the subsequent discovery of severe genetic defects in enzymes involved in homocysteine metabolism as causative factors (4). As the metabolic pathways related to homocysteine became delineated other important polymorphic variants were described, such as the thermolabile variant of the folate metabolizing enzyme, methylenetetrahydrofolate reductase (MTHFR) (5, 6).

The advent of convenient laboratory assays revealed mild to moderate elevation of blood homocysteine concentrations in association with vitamin insufficiencies (i.e., folate, vitamin B12, vitamin B6, and riboflavin), several diseases and disorders, some pharmaceuticals, and lifestyle factors (7). However, in each case, considerable debate still exists concerning the exact contribution of elevated blood homocysteine to disease pathogenesis.

There have also been important recent developments in the closely related metabolic cycles involving folate, vitamin B12, vitamin B6, and riboflavin. For example, the delineation of sub-cellular folate and one-carbon metabolism in mitochondria and the nucleus (8), the importance of formate in said metabolism (9, 10), identification of the transcobalamin receptor by which vitamin B12 is taken up into cells (11), and delineation of the sequence of events underlying intracellular vitamin B12 processing (12–14).

This review describes the basics of homocysteine metabolism and the factors that influence it; considers the diseases, disorders and conditions associated with impaired homocysteine metabolism; and discusses key developments, unanswered questions, controversies, and future research directions. It is intended to provide a starting point and general overview of homocysteine in relation to health and disease for the interested “research novice.” As such, we have endeavored to direct the reader to more comprehensive reviews of specific areas where appropriate.

## Overview of homocysteine metabolism and causes of hyperhomocysteinemia

**Figure 2:**
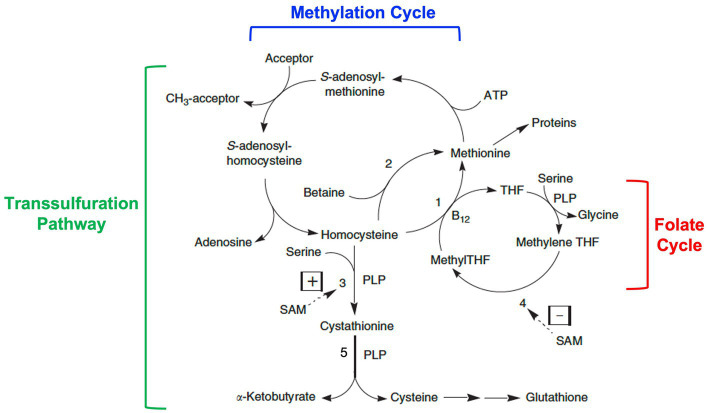
Homocysteine metabolism: interrelationships among the methylation cycle, the transsulfuration pathway, and the folate cycle. Homocysteine is a product of the methylation cycle in which dietary methionine is activated by the addition of an adenosyl group from ATP to form S-adenosylmethionine (SAM). SAM then donates a methyl group to one of a variety of methyl acceptors, such as DNA, RNA, histones, proteins, membrane phospholipids, neurotransmitters, and guanidinoacetate. S-adenosylhomocysteine is produced as a product, which then loses its adenosine group to form homocysteine. Homocysteine can then be converted back to methionine by methionine synthase (1) with methyltetrahydrofolate (MethylTHF) serving as the methyl donor and vitamin B12 serving as a cofactor (occurring in all tissues), or by betaine-homocysteine methyltransferase (2) with betaine (a product of choline metabolism) serving as the methyl donor (occurring only in the liver and kidneys). MethylTHF is produced in the folate cycle by the reduction of methylenetetrahydrofolate (methyleneTHF) catalyzed by the enzyme methylenetetrahydrofolate reductase or MTHFR (4), a FAD (riboflavin)-dependent enzyme. Alternatively, homocysteine can be catabolized through the transsulfuration pathway to cystathionine by the enzyme cystathionine β-synthase (3) and then cysteine by the enzyme cystathionine γ-lyase (5), two vitamin B6 or pyridoxal-5′-phosphate (PLP)-dependent reactions. Further metabolism of cysteine produces the antioxidant glutathione among other products. Whether homocysteine is remethylated back to methionine or catabolized via transulfuration is in part regulated by SAM through allosteric activation of cystathionine β-synthase (3) and allosteric inhibition of MTHFR (4). Modified from Miller (1).

Homocysteine is a non-essential amino acid derived from the metabolism of dietary methionine. Dietary methionine is usually present in about 60% excess over its requirements for protein synthesis. This excess is degraded via the methylation cycle to homocysteine (Figure 2). Subsequently, homocysteine is either remethylated back to methionine or catabolized by transulfuration to cysteine and other downstream metabolites.

In the methylation cycle, methionine is activated by the addition of an adenosyl group from ATP to form S-adenosylmethionine (SAM). SAM is known as the “universal methyl donor” as it serves to provide a methyl group for numerous methylation reactions, including those involving DNA, RNA, histones, proteins, membrane phospholipids, the synthesis of creatine, and the synthesis and metabolism of neurotransmitters. In the process, S-adenosylhomocysteine is formed, which is then further metabolized to adenosine and homocysteine. Homocysteine can then be recycled back to methionine by the enzyme methionine synthase (also known as 5-methyltetrahydrofolate-homocysteine methyltransferase) in a reaction that requires folate in the form of 5-methyltetrahydrofolate as the methyl donor and vitamin B12 in the form of methylcobalamin as a cofactor. This reaction occurs in all cells. An alternative folate- and vitamin B12-independent reaction utilizes betaine (a metabolite of choline) as the methyl donor to convert homocysteine to methionine. This reaction only occurs in the liver and kidneys and is catalyzed by the enzyme betaine-homocysteine methyltransferase.

Alternatively, homocysteine is catabolized via the transsulfuration pathway by first condensing with the amino acid, serine, to form cystathionine, which is then cleaved to form cysteine and α-ketobutyrate. These reactions are catalyzed by the enzymes, cystathionine β-synthase and cystathionine γ-lyase respectively, both of which require pyridoxal 5′-phosphate (the active form of vitamin B6) as a cofactor. Further metabolism of cysteine leads to the formation of the tripeptide glutathione (a key intracellular antioxidant) or conversion to taurine, sulfate, and pyruvate.

The activities of remethylation and transulfuration are closely co-ordinated and the two pathways can be considered to be in competition for available homocysteine (15). Reduction of activity in one pathway will lead to more effective use of homocysteine by the other pathway. Changes in dietary methionine will lead to changes in the cycling of homocysteine via the methylation pathway. When dietary methionine is halved, the number of cycles per homocysteine moiety doubles. Conversely, when dietary methionine is increased homocysteine cycling decreases (16, 17).

Whether homocysteine is conserved by remethylation back to methionine or degraded through transsulfuration depends on intracellular levels of SAM (16). This is based on the role of SAM as both an allosteric activator of cystathionine β-synthase and an allosteric inhibitor of MTHFR, the enzyme that produces 5-methyltetrahydrofolate (Figure 2). When cellular SAM concentrations are high, as would be expected after a meal containing protein (and thus methionine), SAM activates cystathionine β-synthase and inhibits the synthesis of 5-methyltetrahydrofolate by MTHFR. This promotes homocysteine catabolism and diminishes homocysteine remethylation. When cellular SAM concentrations are low the synthesis of 5-methyltetrahydrofolate by MTHFR will proceed uninhibited, whereas cystathionine β-synthase activity will be diminished. This results in the conservation of homocysteine for methionine synthesis. In this way, SAM serves as a “sensor” of dietary methionine such that homocysteine is recycled to reform methionine when dietary intake is low and is catabolized when dietary intake is high.

An implication of this allosteric regulation is that elevation of homocysteine in the blood, called “hyperhomocysteinemia,” stems from conditions in which the cell is no longer capable of coordination between the two pathways (16). For example, impairment of the remethylation pathway will lead to decreased intracellular SAM concentrations, which thwarts the induction of transsulfuration and diminishes catabolism of excess homocysteine. In this way, both remethylation and catabolism of homocysteine are impaired, and the accumulated intracellular homocysteine is transported into the blood causing hyperhomocysteinemia. Impairment of remethylation occurs when there is dietary deficiency of folate or vitamin B12, or defects in MTHFR, methionine synthase, or one of the enzymes required for the formation of methylcobalamin (7).

In contrast, but with ultimately the same result, impairment of transsulfuration will lead to increased intracellular SAM concentrations and consequent inhibition of MTHFR, which reduces the formation of 5-methyltetrahydrofolate for remethylation and inhibits recycling of homocysteine through its conversion to methionine (16). Again, both pathways are impaired, and the accumulated homocysteine is transported into the blood. Impairment of transsulfuration occurs when there is dietary deficiency of vitamin B6, or a defect in cystathionine β-synthase.

While this SAM-mediated allosteric control system is present in many organs, especially the liver, its effect on the methylation cycle in the central nervous system (CNS) may be limited. Cystathionine γ-lyase is absent in the CNS. Thus, under circumstances of SAM excess homocysteine cannot be catabolized to cysteine and accordingly accumulates as cystathionine in brain (18). Also, there is no alternative method whereby homocysteine can be remethylated to methionine since betaine-homocysteine methyltransferase is absent in the brain. Thus, it has been pointed out that the brain is totally reliant on methionine synthase activity to remethylate homocysteine (19).

There is also evidence for additional regulatory mechanisms for the co-ordination of remethylation and transsulfuration. Cystathionine β-synthase is unique in that it is dependent not only on pyridoxal 5′-phosphate as a cofactor, but heme as well. It has been found that the heme oxidation state influences the enzyme’s activity (20). Under oxidizing conditions cystathionine β-synthase activity is increased *in vitro*. It is suggested that this leads to an increase in the synthesis of cysteine and then the antioxidant glutathione, and thus represents a physiological feedback mechanism for limiting oxidative stress.

Hormones also play a regulatory role. Blood homocysteine levels tend to rise in women after menopause, indicating that estrogen regulates homocysteine metabolism (21). This is supported by observations that blood homocysteine decreases in individuals undergoing male to female transition, and vice versa in those undergoing female to male transition (22). The mechanism by which estrogen promotes homocysteine metabolism is not well understood. In hypothyroidism, blood homocysteine concentrations are increased and decrease after the initiation of thyroid hormone supplementation (23). As for estrogen, it is not clear how thyroid hormone affects homocysteine metabolism. In type 1 diabetes, blood homocysteine tends to decline due to reduced insulin production. This is believed to occur because insulin is an inhibitor of cystathionine β-synthase expression, and in the absence of insulin there is higher catabolism of homocysteine through cystathionine and cysteine synthesis (24). Here it should be noted that homocysteine levels are often elevated in diabetes patients. This likely occurs in later stages of the disease when renal function is affected (25). The kidneys are a major organ for homocysteine metabolism, and renal insufficiency or failure leads to hyperhomocysteinemia (26, 27).

## Homocysteine and inborn errors of metabolism

Often, what is known about biochemical pathways originates from observations of the “natural experiments” that are inborn errors of metabolism. This is certainly true for homocysteine metabolism and the genetic disorder known as homocystinuria. From the previous section, it follows that inherited disorders leading to dramatic elevations of homocysteine in the blood and urine derive from genetic defects in homocysteine transsulfuration or re-methylation, or in the transporters and enzymes involved in the delivery and metabolism of the key cofactors required for these metabolic pathways (specifically folate and vitamin B12).

Severe cases of homocystinuria are typically caused by autosomal recessive genetic defects and exhibit characteristic clinical features including ocular lens dislocation, marfanoid features and other skeletal abnormalities including osteoporosis, intellectual disability, and thromboembolic disease, the latter frequently being the cause of premature death in affected individuals (28). The known recessive genetic defects that cause homocystinuria are summarized here:

### Cystathionine β-synthase deficiency

Homocysteine, in its oxidized disulfide form (Figure 1), was first detected in the urine of two siblings with intellectual disability in Ireland in 1963 (29). Shortly afterwards the cause of the disease was identified as impaired cystathionine β-synthase enzyme activity (30). Treatment options include dietary methionine restriction (to reduce the overall burden of homocysteine) and cysteine supplementation (to compensate for impaired synthesis of cysteine), as well as vitamin B6 administration (to maximize residual cystathionine β-synthase activity). Careful monitoring and maintenance of folate and vitamin B12 status is also important to maximize remethylation of homocysteine back to methionine. Betaine supplementation is also common to promote the folate- and vitamin B12-independent conversion of homocysteine to methionine in the liver and kidney.

### Inherited disorders of vitamin B12 absorption, transport, and metabolism

The years following the discovery of homocysteine brought considerable developments in our understanding of the metabolic role of vitamin B12 (also known as cobalamin). A form of anemia associated with stomach degeneration (pernicious anemia or PA) was fatal until 1926 when two American physicians, Minot and Murphy, described a curative raw liver diet (31). For the next two decades, liver was the main source of this unknown “extrinsic factor”. In 1929 Castle observed that gastric juice contained a protein he called “intrinsic factor” (IF), which enhanced the clinical effects of extrinsic factor (32). In 1948, two independent teams in the USA (33) and England (34) isolated the mysterious extrinsic factor in crystalline form. Folkers called it “vitamin B12.” Its production on an industrial scale in the early fifties enabled its worldwide medical application to treat PA.

As described above, vitamin B12, in the form of methylcobalamin, is an essential co-factor for methionine synthase and the remethylation of homocysteine (Figure 2). Inborn errors can occur in the absorption and transport of vitamin B12, as well as its intracellular delivery and metabolism (35). Although disorders of absorption and transport are associated with hyperhomocysteinemia, this is not as marked as that arising from intracellular defects.

Three proteins are involved in the absorption and transport of vitamin B12 – haptocorrin, intrinsic factor, and transcobalamin. Haptocorrin deficiency is rare but relatively benign with no reports of elevated homocysteine in these patients (36). Defective intrinsic factor synthesis arises from mutations in the *GIF* (gastric intrinsic factor) gene and usually presents during infancy, after vitamin B12 stores are depleted (35). Features resemble adult PA, an autoimmune disorder associated with autoantibodies to gastric parietal cells or gastric IF, and it is associated with both elevated methylmalonic acid (MMA) and hyperhomocysteinemia.

Hyperhomocysteinemia and elevated MMA can also arise from Imerslund-Gräsbeck syndrome—a rare autosomal recessive disorder caused by mutations in either the *CUBN* or *AMN* gene responsible for the synthesis of IF receptors (cubam) in the ileum (distal region of the small intestine) (35). It is accompanied by a mild proteinuria in ∼50% of cases, due to renal cubam receptor expression.

Transcobalamin deficiency is a rare autosomal recessive disorder arising from mutations in the transcobalamin (*TCN2*) gene (35). Serum vitamin B12 levels are normal, due to the presence of vitamin B12 on haptocorrin, but it is associated with both elevated blood homocysteine and MMA. It usually presents in infancy with a severe phenotype including failure to thrive, weakness, anemia and pancytopenia, and requires life-long treatment with high doses of vitamin B12 (37).

The transcobalamin receptor (CD320) was recently identified (11) though deficiency appears to be extremely rare, with only a dozen cases recorded to date (38). Affected individuals have some degree of methylmalonic aciduria and homocystinuria, although not as high as in patients with inherited disorders of intracellular vitamin B12 metabolism (12).

**Figure 3:**
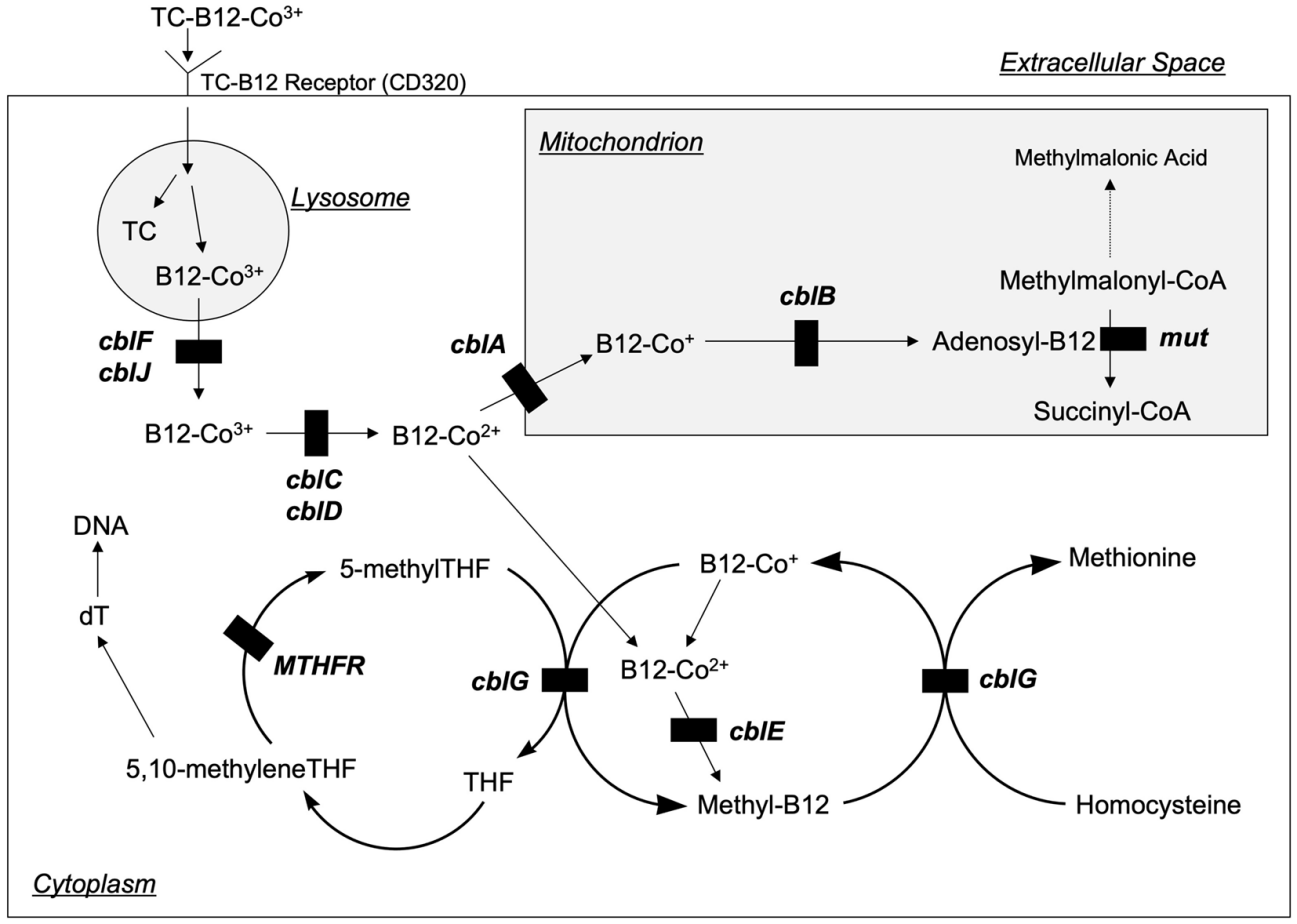
Intracellular vitamin B12 metabolism. Vitamin B12 is taken up by cells bound to transcobalamin (TC) via the transcobalamin receptor (also known as CD320). The TC and B12 are disassociated in the lysosome, and then B12 is transported into the cytoplasm where it is metabolized to form methylcobalamin (Methyl-B12) or into the mitochondria where it is metabolized to form 5-deoxyadenosylcobalamin (Adenosyl-B12). The transport and metabolism of B12 is carried by a variety of proteins that are designated as cblA—cblG, cblJ, and mut (not pictured are two additional proteins, cblK and cblX, which act in the nucleus). Genetic defects in several of these proteins inhibit B12 processing and can lead to homocystinuria. Modified from Rosenblatt (39).

An important advance related to homocysteine has been the detailed characterization of intracellular vitamin B12 metabolism (Figure 3) (12–14). These disorders comprise a total of 10 clearly recognized defects designated cblA-cblX, defined by means of *in vitro* somatic complementation analysis (12). In the cblC, cblD, cblF, cblG, cblJ, and cblX group of disorders, the synthesis of both adenosylcobalamin and methylcobalamin are affected, leading to combined methylmalonic aciduria and homocystinuria. In cblA and cblB disorders, only the synthesis of adenosylcobalamin is impaired, resulting in methylmalonic aciduria but normal homocysteine concentrations, whilst the cblE disorder results in isolated homocystinuria.

The cblC defect, although the commonest, is still rare, arising in approximately 1/200,000 births (12). Clinical manifestations often appear in infancy but can occur in later life. Typical symptoms associated with early-onset disease are feeding difficulties, failure to thrive, hypotonia, seizures, pigmentary retinopathy, developmental delay, and macrocytic anemia. Late-onset disease is frequently accompanied by neurological dysfunction, including cognitive decline, psychosis, or dementia. In comparison with early-onset patients, late-onset patients have better survival and response to therapy.

Treatment of inherited disorders of vitamin B12 absorption, transport, uptake and metabolism typically includes regular, high-dose intramuscular injections of vitamin B12 in the form of cyanocobalamin (standard in the United States), hydroxycobalamin (standard in Europe), or increasingly methylcobalamin. Alternatively, daily high-dose oral supplements may be used that promote vitamin B12 absorption through passive diffusion along the length of the gastrointestinal tract (40). For a detailed discussion of vitamin B12 intracellular disorders and response to treatment the reader is referred to several reviews on this subject (12–14).

### Methylenetetrahydrofolate reductase deficiency

As described above, the methyl group required for remethylation of homocysteine is provided by 5-methyltetrahydrofolate formed by MTHFR (Figure 2). Hence, MTHFR deficiency results in hyperhomocysteinemia with clinical features similar to cystathionine β-synthase deficiency. At least 40 rare MTHFR gene variants have been found in people with decreased or no working enzyme (41). Treatment options include high-dose betaine supplements started early in life that maximize the conversion of homocysteine to methionine via the folate- and vitamin B12-independent pathway. Supplements of folic acid and vitamin B12 are often prescribed to maximize residual MTHFR activity and promote methionine synthase activity. As the product of MTHFR, supplements of 5-methyltetrahydrofolate may be better than folic acid in treating this cause of homocystinuria.

Notably, a far more common variant in MTHFR, the 677C- > T substitution (which causes an alanine to valine change in the amino acid sequence of the enzyme) results in increased plasma homocysteine (5, 6). The elevation of homocysteine is accentuated when the affected individual is low in folate or riboflavin, the latter serving as a cofactor for the MTHFR enzyme in the form of FAD. Homozygosity for 677 T variant is estimated to occur in 10–15% of the general population, with lower prevalence in individuals of black African descent, and higher prevalence in Hispanic and some Asian populations (42). The homozygous variant is associated with increased risk of coronary artery disease, venous thrombosis, and in particular cerebral infarction and stroke (43). It is also associated with increased blood pressure and risk of hypertension, which is responsive to riboflavin supplements (44).

## Homocysteine and vascular disease

The pathologist, Kilmer McCully, proposed the “homocysteine theory of atherosclerosis” in the 1960’s after comparing the vascular pathologies of two young children who died of thromboembolic disease. The two had different genetic causes of homocystinuria, but exhibited similar patterns of extreme elevations of homocysteine in the blood and urine (45). Later, in the 1980s, the development of high-pressure liquid chromatography methods allowed for rapid determination of homocysteine concentrations in 1000’s of samples, thus paving the way for epidemiological studies that revealed significant associations between relatively small elevations of homocysteine in blood and cardiovascular, cerebrovascular, and peripheral vascular diseases (7, 46). However, while B vitamin supplements effectively lower homocysteine concentrations, many intervention trials failed to show a reduction in vascular disease risk (47). These confounding observations suggest that homocysteine may be a biomarker of vascular risk but not a causative factor or target for intervention.

However, in a recent comprehensive and thoughtful review, Smith and Refsum argue that there is indeed a benefit to lowering homocysteine with B vitamin supplements, but that the efficacy depends on the characteristics of those being treated before supplementation is initiated (48). First, there must be evidence of elevated homocysteine or low B vitamin status. Under conditions of non-elevated homocysteine and adequate B vitamin status, there is little or no expectation that additional supplementation will further reduce disease risk. Second, an individual must be at risk for the outcome of interest over the supplementation period, otherwise, the intervention will have nothing to prevent. Third, consideration must be paid toward potential confounding factors that can modify the effects of the supplements and homocysteine-lowering. Moreover, Smith and Refsum argue that even if sub-group analyses were not pre-specified in a given study, such analyses can be informative and hypothesis-generating, and should not be dismissed.

These factors are especially true for the association between hyperhomocysteinemia and stroke. Many trials have assessed the effect of B vitamin supplements and homocysteine-lowering on stroke outcomes, with mixed results. An informative example worth considering is the HOPE-2 trial (49). In this study B vitamins did not influence the overall numbers of cardiovascular events despite effectively lowering blood homocysteine concentrations, though there was a significant reduction in the risk of stroke. The authors of the trial did not consider the effect on stroke outcomes to be valid because they opined that the pathogenic mechanisms underlying myocardial infarction and stroke were essentially the same and therefore an effect of homocysteine-lowering should have been observed for both vascular outcomes. This conclusion was later opposed by Spence (50), as there are indeed significant differences in the pathogenesis and etiology between myocardial infarction and stroke. When the HOPE-2 trial (51), and other major trials such as VISP and VITATOPS (52), were re-analyzed, specific subgroups were identified that were most likely to benefit from B vitamin supplementation in reducing stroke risk, including (1) adults less than 69 years of age, (2) those not exposed to folic acid fortification, (3) those with elevated homocysteine or cholesterol, and (4) those who were not taking anti-platelet or lipid-lowering drugs. This illustrates that for almost any treatment, including B vitamin supplements, “one size does not fit all,” and that lack of an overall effect in a study population does not necessarily mean there are not individuals within that population who might benefit. These are important considerations that can guide clinicians and their patients on whether B vitamin supplements could be beneficial.

## Homocysteine, age-related cognitive impairment and dementia

Technological advances in homocysteine measurement not only enabled large-scale epidemiological studies of associations with vascular disease, but also heralded an avalanche of literature describing its association with age-related cognitive impairment, including Alzheimer’s disease (AD) and dementia (53, 54). Credible potential mechanisms underlie the association (see below), which fulfils Bradford-Hill’s criteria suggesting causality (55).

Similar to the points described above regarding the efficacy of B vitamins in reducing stroke risk, baseline characteristics of individuals greatly influence whether reduced risk of age-related cognitive decline and Alzheimer’s disease/dementia can be achieved by such intervention. Lowering homocysteine with high dose B vitamins is effective in slowing cognitive decline and brain atrophy (56, 57), though this effect is dependent on baseline homocysteine concentrations. In other words, metabolic evidence of B vitamin deficiency must be present at the beginning of the intervention. The VITACOG study (58) was well designed, focusing on patients with mild cognitive impairment but not yet diagnosed with dementia. Thus, the participants were experiencing cognitive decline, the trajectory of which could conceivably be altered by interventions such as B vitamins and homocysteine lowering. Contrast the Alzheimer’s Disease Co-operative Study (59), in which the participants had already progressed to moderate to severe dementia. Notably, no benefit of homocysteine lowering was observed in this trial. This makes intuitive sense in that, once neurodegeneration progresses too far, interventions such as B vitamins are likely to be ineffective. Via similar logic, interventions in individuals who do not yet exhibit age-related cognitive impairment (either dementia or mild cognitive impairment) may also yield no effects of B vitamins/homocysteine lowering as it is difficult to prevent something that is not happening (55).

With that said, the biological mechanisms by which homocysteine-lowering with B vitamins might prevent cognitive decline and dementia have not yet been clearly delineated. Importantly, if B vitamin supplementation does slow or prevent age-related cognitive decline, it is difficult to determine if this is directly due to homocysteine-lowering or to some other biochemical or physiological effects of one or more of the B vitamins. These issues are discussed further below (*see* “*Current issues and controversies*”).

## Neural tube defects and other pregnancy outcomes

Neural tube defects (NTDs), including anencephaly, spina bifida, and related pathophysiological developmental defects, occur if the neural tube does not completely close between 3- and 4-weeks post-conception. They are caused by both genetic and environmental factors. Data from the “Dutch hunger winter” of 1944–45 suggested a nutritional basis; a peak incidence of spina bifida was noted amongst men conceived between February and March 1945, i.e., the depths of the Dutch famine during World War II (60). In 1964, Hibbard noted the role of folic acid in relation to placental abruption and abortion (61). Smithells et al. (62) later found that the use of multi-vitamins containing folic acid during peri-conception conferred protection against NTDs. The efficacy of folic acid in preventing NTDs was confirmed by the MRC Vitamin Study Research Group in 1991 (63). These and other findings (64) led to the recommendation in 1992 by the United States Centers for Disease Control and Prevention that women of childbearing age consume 0.4 mg/day of folic acid to prevent NTDs (65), and the subsequent mandate in 1996 by the United States Food and Drug Administration that all cereal grain products be fortified with folic acid (66). This fortification program has been highly successful, with reductions in incidence in the United States variously estimated to be 19–40%, depending on the type of NTD and how incidence was counted (67). Today, more than 80 countries around the world have mandatory folic acid fortification (68).

Because folate status is a major determinant of blood homocysteine concentrations, the possible contribution of elevated homocysteine to the pathogenesis of NTDs has been postulated. Eskes hypothesized that hyperhomocysteinemia, even with normal folate levels, could be a toxic agent for the developing embryo (60). Hyperhomocysteinemia is associated with early pregnancy losses (69), and many studies also report that a moderate elevation in early pregnancy is inversely associated with birth weight and predictive of intrauterine growth retardation [See (70) for review]. Notably, low vitamin B12 status is also a risk factor for NTDs (71), further suggesting that homocysteine toxicity may be a factor in their pathogenesis.

## Current issues and controversies

### Excess folic acid exposure and exacerbation of vitamin B12 deficiency

Homocysteine lies at the intersection of folate and vitamin B12 metabolism, and the biological and clinical interaction between these two vitamins has been a subject of considerable interest for decades. Perhaps surprisingly, there remain unresolved issues.

From the mid 1940s to mid 1950s several clinical studies reported neurological harm in PA patients inappropriately treated with folic acid (72, 73). It was known that folic acid corrects the anemia of vitamin B12 deficiency but it was not immediately appreciated that neurological changes and permanent nerve damage occurred if the underlying vitamin B12 deficiency remained untreated (74). This is the essence of the concept that folic acid “masks” vitamin B12 deficiency. Notably, some suggested that folic acid supplements not only masked vitamin B12 deficiency, but even exacerbated or accelerated it. It was also noted that vitamin B12 levels fell in epileptic patients treated with folic acid alongside their anticonvulsant medication (75). Subsequently, the inappropriateness of treating PA with folic acid was recognized and the practice was discontinued. However, an accepted explanation for the effect of folic acid on the progression of neurological damage remained obscure.

However, this putative “interaction” between vitamin B12 and folate has important implications for public health, especially in the era of food-folate fortification. The rationale for many countries to introduce such fortification was to reduce the incidence of NTDs (*see above*), and it has proved remarkably successful (76). However, mandatory fortification coupled with personal consumption of folic acid-containing supplements exposes a significant proportion of such populations to amounts of folic acid well above the recommended upper tolerable intake level established by the Institute of Medicine (77). Concerns have arisen regarding the potential toxicity of such exposure. These include associations with an increased incidence of colorectal and breast cancers, autism, and cognitive function (77), although controversy exists regarding causality (78).

Of particular note is that several cohort studies have found that high folate status (typically indicated by elevated serum folate concentrations) is associated with apparently exacerbated outcomes in individuals with low vitamin B12 status (typically indicated by low serum B12 concentration or elevated MMA concentrations), including higher risk of anemia and cognitive impairment (79), and accentuated elevations of blood homocysteine and MMA (80, 81). These findings have resurrected the possibility that high folate status, or perhaps more specifically exposure to excess folic acid, may be harmful to people with low or deficient B12 status. This issue is very controversial, however, because most of the evidence is cross-sectional and associative, and no mechanism has been empirically determined to explain this low B12/high folate interaction. Notably, no effect of high-dose folic acid (5 mg/day) for 3-months on blood MMA concentrations was observed in a cohort of middle-aged individuals (82). This may indicate that the association is not causal or that the effect is age-dependent (most of the cohort studies included older adults) or only observed in individuals with a more deficient B12 status.

With that said, several mechanistic hypotheses have been put forward to explain the putative low B12/high folate interaction. One hypothetical explanation is an adverse oxidative effect of unmetabolized FA (UMFA) on vitamin B12 homeostasis (80). Alternatively, people with existing low B12 status might fail to synthesize polyglutamated intracellular folate—an essential prerequisite for its cellular retention—hence leading to a state of elevated serum folate in the face of low cellular and serum vitamin B12, and accounting for the observed cross-sectional association between elevated serum MMA and folate (83). A more recent hypothesis postulates that high-dose folic acid redirects the active form of vitamin B12 in serum, i.e., holotranscobalamin, either toward the bone marrow to support reticulocyte synthesis that is induced by folic acid in B12-deficient patients, or into the urine in which it is excreted instead of being taken up and recycled into circulation through the kidneys (84). The diversion of holotranscobalamin to the bone marrow or the urine depletes the liver, brain, and other tissues of vitamin B12, thus exacerbating neurological manifestations of deficiency and elevations of homocysteine and MMA in the blood. Circumstantial evidence for this hypothesis comes from one cohort study in which the combination of low B12 and high folate status was associated with lower serum holotranscobalamin concentrations than when B12 was low but folate was not elevated (81). This hypothesis remains to be tested empirically.

### High serum cobalamin (“hypercobalaminemia”)

Maintaining adequate vitamin B12 status through diet and supplementation (as needed) is unquestionably important for health. Until recently, also unquestioned was that exposure to excess vitamin B12 did not have any negative effects. There is no established upper tolerable intake level for B12 and doses 1000s of times the RDA level are generally well tolerated. However, a small number of recent reports have found associations between elevated serum levels of B12 and various outcomes, including overall mortality (85). However, what is unclear from these studies is whether elevated serum B12 is a cause of increased mortality and related morbidities (e.g., cardiovascular disease, cancer), a consequence of other conditions associated with increased mortality (e.g., hematological, liver or kidney disease), or simply coincidental to these conditions. In addition to excess intake, high serum B12 concentrations can also arise from increased production of carrier proteins (transcobalamin and haptocorrin) or decreased renal or hepatic clearance (86). Hence, a high level can indicate one of several conditions including hematological malignancies, renal failure and hepatic diseases including hepatocellular carcinomas. Corcoran et al. (87) also found a weak association between serum B12 and C-reactive protein concentrations on admission to an intensive care unit. However, the potential of B12 status as an “acute phase reactant” requires further study; falsely elevated levels can also arise due to the formation of immune complexes with B12-binding proteins (88). At this time, the evidence that excess B12 may be harmful is limited and largely circumstantial. The limited nature of the evidence is an important consideration for individuals who truly require B12 supplementation, such as those with PA and other B12 malabsorption conditions, and those that follow vegan and vegetarian diets. Such individuals should not eschew B12 supplementation and thus trade a fair certainty of benefit to prevent a vague and not firmly established risk of harm. Nonetheless, future research should explore this issue further.

### Homocysteine and cognition

As discussed above, elevated blood homocysteine levels might arise for many different reasons, e.g., dietary and genetic factors. Similarly, there are multiple mechanisms by which elevated homocysteine could adversely affect cognitive processes (89). These include altered choline metabolism, changes in neurotransmitter metabolism, defective DNA methylation with consequent poly (ADP-ribose) polymerase (PARP) overactivation and PARP-controlled cell death, excitotoxicity, up-regulation of re-entry to the cell division cycle, hypomethylation of myelin lipids and myelin basic protein, white matter changes, and the formation of homocysteine thiolactone and subsequent protein homocysteinylation (89).

Any of these mechanisms could contribute, to a variable extent, to several neurodegenerative diseases. However, disturbed one-carbon metabolism might also be closely linked with two key AD features—amyloid plaques and tau tangles.

*Tau* stabilizes microtubules. In healthy neurons microtubules form “railway-like” structures, which guide nutrients and other molecules down the axon; *tau* supports these structures. It is normally phosphorylated, but in AD and other neurodegenerative diseases it appears to be overly phosphorylated (90). This causes its aggregation into tangles, and this microtubule transport system disruption impairs neuronal function. *Tau* hyper-phosphorylation occurs due to an imbalance between “kinase” and “phosphatase” enzyme activity. Kinase adds phosphate groups to *tau*, but the phosphatase PP2A removes them. PP2A activity declines with age but it is also reduced in the hippocampus of AD patients. Methylation of PP2A subunits are critical for its activity, and it is suggested that decreased PP2A methylation might link hyperhomocysteinemia to tangle formation (91).

The other hallmark feature of AD—the amyloid plaque—forms from β-amyloid (Aβ), a small peptide derived from cleavage of amyloid precursor protein (APP). Its physiological function remains unknown. It is synthesized in the endoplasmic reticulum, passes through the golgi complex, and is inserted into intracellular vesicle membranes. APP is processed by α- and β-secretases. Alpha-secretase cleavage results in secreted forms of APP (because α-secretase cleaves in the middle of Aß, it precludes the release of potentially amyloidogenic Aβ). β-secretase also releases secreted APP but leaves behind a membrane-spanning fragment containing intact Aβ. This is processed further by γ-secretase resulting in the release of intact Aβ with the potential to form amyloid plaques.

Homocysteine also activates several genes related to endoplasmic reticulum stress. One of these codes for the “Herp” protein. This suggests a link between homocysteine and the generation of plaques. Presenilin 1 and 2 constitute the catalytic subunit of γ-secretase activity and are therefore responsible for the final step in β-amyloid biogenesis. Sai et al. (92) found that Herp interacts with presenilin 1 and 2 such that a high expression of Herp in cells increases the generation of Aβ. Herp therefore provides a link between homocysteine and this other key feature of AD pathology.

Despite the above mechanisms it is also possible that, to some extent, the association between AD and homocysteine simply reflects co-existing vascular disease rather than an association with “pure” AD pathology. Many individuals with plaques and tangles do not become demented—the *clinical* expression of the disease seems to be partly determined by co-morbid conditions such as brain infarcts and small vessel disease.

In addition, vascular endothelium and neuronal tissue are both particularly sensitive to oxidative stress, and it is possible the observed relationships might reflect the effects of redox status on homocysteine metabolism. To fully understand this, one needs to consider the mechanism of the methionine synthase reaction in relation to vitamin B12 and its various oxidation states.

Vitamin B12 comprises a cobalt atom at the center of a tetrapyrrole ring with a variable upper axial ligand, such as a methyl, adenosyl, hydroxo, or cyano group (93). Dimethylbenzimidazole is bonded to one pyrrole and usually coordinates to cobalt as the lower axial ligand. The cobalt atom exists in three different oxidation states. In the cob(III)alamin state it is co-ordinated to the pyrrole ring as well as the upper and lower ligands. When cobalt is coordinated to the pyrrole ring alone, it is in a cob(I)alamin state. Removal of one or other axial ligands leaves cobalt in an intermediate cob(II)alamin state. In the primary turnover cycle of the MS reaction homocysteine reacts with the methyl group of methionine synthase-bound methylcobalamin to produce methionine and an unstable intermediate form, cob(I)alamin. Cob(I)alamin then reacts with 5-methyltetrahydrofolate to generate tetrahydrofolate and regenerate methylcobalamin. Vitamin B12 shuttles between methylcobalamin and cob(I)alamin states. However, cob(I)alamin is occasionally deactivated by oxidation to cob(II)alamin. Return to the primary turnover cycle requires a reactivation step in which SAM is the methyl donor. This deactivation and reactivation occur every few hundred cycles *in vitro*. AD and age-related oxidative stress likely augment this process (94). In addition, the homocysteine binding site of methionine synthase can also be oxidized, and folate itself can undergo irreversible oxidation. It is also likely that intracellular reduction of vitamin B12 to its active state, which requires reduced glutathione (95), might also be compromised under such conditions.

Thus, homocysteine probably compromises neuronal homeostasis by multiple, divergent routes. Assessing the relative importance of these mechanisms will be an exciting and hopefully fruitful aspect of future research. This is discussed further below.

## The next 90 years: what might the future hold?

*“Prediction is very difficult, especially if it’s about the future!”* (Niels Bohr).

It is inconceivable that Butz and du Vigneaud could have anticipated the relevance of their discovery in relation to so many diverse diseases and disorders. Similarly, it is impossible to accurately predict developments in relation to homocysteine research over the next 90 years. However, it is likely that we will witness further advances in diagnostic techniques and point-of-care testing which may well be applicable to homocysteine. For example, it is not inconceivable that eventually micro- or nano-fluidic technology and other future technologies will deliver the “lab-on-a-chip” that Theranos recently scandalously failed to deliver (96). Given such advances, one could imagine developing metabolomic profiles, backed up with large databases and artificial intelligence, that could be used for “point-of-care” testing, health screening and diagnosis. Homocysteine, by virtue of its relationship with cardiovascular, cerebrovascular, and thromboembolic diseases, could well comprise part of that profile. Notably, assessment of homocysteine could serve as an early biomarker of disease risk, even before clinical signs and symptoms are manifested, thus potentially allowing for early intervention and greater potential for amelioration or outright prevention.

It is also hoped there will also be improvements in functional biomarkers to assess both folate and vitamin B12 status (78). For example, if markers of vitamin B12 status do not show a “clear-cut” deficiency, Fedosov et al. (97) have suggested using a “combined” indicator of vitamin B12 status (cB12) based on a mathematical model combining two to four biomarkers (serum total vitamin B12, holotranscobalamin, homocysteine and MMA). By combining the biomarkers, assessment of vitamin B12 status gains both specificity and sensitivity, and thus improves diagnostic accuracy and precision. The current drawbacks of this strategy are cost and lack of availability of all tests in routine clinical practice. Diagnostic advances and cost reduction should lead to its more widespread adoption.

Along similar lines, the future may bring increased interest in assessing the extent to which people are exposed to excess folic acid. The term “folate” refers to a family of related molecules that are interconvertible and have biochemical functions. Folic acid is a synthetic, unnatural form of folate, which nonetheless is capable of being absorbed, transported, taken up by cells, and converted (by the action of the enzyme dihydrofolate reductase) to the active forms of folate. This is why it is effective as a supplement and fortificant. However, the conversion of folic acid to active forms of folate is relatively slow in human cells (98), and exposure to high amounts of folic acid through supplements and fortified foods leads to the measurable appearance of unmetabolized folic acid in the blood. As described above, this may have health consequences, such as exacerbation of B12 deficiency (84). This raises the question of whether specifically measuring folic acid in blood samples (as opposed to the total of all forms of folate) might provide additional information regarding health risks. This remains to be explored.

Inter-individual variability in homocysteine, due to genetically determined metabolic heterogeneity, will likely become a fruitful area of research. A better understanding of this variability, due to diagnostic improvements, advances in computational and mathematical modeling of biological systems and, perhaps, the use of artificial intelligence systems will allow accurate prediction of these variations. In people in whom such metabolic inefficiencies are identified, clinicians could then recommend specific diets, supplements, medical foods, or even pharmaceutical interventions to optimize organ function. Although not simply related to homocysteine of course, this approach is the aim of the emerging discipline of “precision nutrition” (99).

An important component of such metabolic heterogeneity is the presence of single-nucleotide polymorphisms (SNPs) in the various genes related to enzymes involved in homocysteine metabolism. The representative example of this is the MTHFR C677T polymorphism cited above (5, 6). Individuals homozygous for the variant form (677TT) have an MTHFR enzyme with reduced affinity for its substrate (methylenetetrahydrofolate) and its riboflavin cofactor (FAD). Based on basic Michaelis–Menten enzyme kinetics, by supplying the extra substrate and cofactor (i.e., folate and riboflavin) the reduced affinity can be overcome and the consequences of having the variant can be alleviated, e.g., reduction in elevated homocysteine, reduction in blood pressure, etc.… This is the very definition of “personalized nutrition,” in this case an increased requirement of two essential nutrients based on a common genetic difference. It also demonstrates that at its essence, nutrition is “applied biochemistry” and that we are all biochemists conducting biochemical experiments every time we eat!

Another area that is pertinent to the concept of precision nutrition is “epigenetics.” This refers to the chemical modification of DNA and associated histones that control, among other processes, whether individual genes are expressed. The classical chemical modification of DNA and histones in this regard is methylation, and the methyl groups required for such methylation come from the diet and one-carbon metabolism. Thus, nutrients involved in one-carbon metabolism (folate, vitamin B12, vitamin B6, riboflavin) putatively affect epigenetic programming (100). This is particularly important during early development when epigenetic programming may determine the health course of an individual throughout life. Bringing this back to homocysteine, it is known that when homocysteine is elevated in the blood, its precursor, S-adenosylhomocysteine, is elevated intracellularly. S-adenosylhomocysteine is a feedback inhibitor of SAM-dependent methylation reactions, and thus hyperhomocysteinemia may affect epigenetic programming as well as other methylation-dependent processes.

In recent years there is an appreciation of the advantages of involving patients in healthcare decisions, in contrast to the traditional “paternalistic” approach to medicine. For example, in relation to homocysteine, this has led to an improved awareness of vitamin B12-related symptomatology as well as highlighting patient frustration regarding treatment options for PA (101, 102). As a result, patients have helped define important unanswered research questions. These include: whether a more accurate test can be developed to diagnose PA; whether an individual’s vitamin B12 requirements change over a lifetime; what are the most effective ways to replenish vitamin B12 stores; why do certain individuals with PA require vitamin B12 injections at different time intervals; and why do some patients experience residual symptoms after treatment? (see https://www.jla.nihr.ac.uk/priority-setting-partnerships/pernicious-anaemia/top-10-priorities.htm). It is possible that adopting such an approach could yield similar valuable insights into folate deficiency and other homocysteine-related disorders.

It is likely that the coming decades will also witness considerable advances in our understanding of the biological processes underpinning memory, and cognition in general. Regarding homocysteine, a key issue will be to assess the relative importance and contribution of the various proposed mechanisms (see above). It has also been noted that it can be difficult to disassociate “homocysteine-specific” effects from “vitamin-lowering” (i.e., high dose B-vitamin) effects (103). Disentangling the effects of “homocysteine lowering” *per se* might prove difficult. One possibility is to consider trials of homocysteine lowering by other means, such as the use of N-acetylcysteine (104).

Given the current unresolved issue regarding a potential low B12/high folate interaction (see above), a case has been made for at least considering vitamin B12 food fortification, although randomized clinical trials are required to fully justify this (105). Nevertheless, it is an issue that will likely be important to address in coming decades. Of note, there are growing calls to move from animal-based diets to more plant-based diets for their putative benefits to both humans and animals, as well as limiting the production of greenhouse gas emissions produced by the animal agriculture industry. This is likely an important, if not necessary, fundamental change in how we feed ourselves, but it is recognized that this could increase the prevalence of vitamin B12 deficiency because the vitamin is not found in plant-based foods (106, 107).

Last, it is important to note that evidence for many of the diverse disorders associated with an elevated homocysteine concentration are based on cross-sectional observational studies. It remains to be seen whether properly conducted randomized clinical trials will strengthen or weaken these associations. Even if such associations are not found to be causal, a plausible explanation for the association itself could yet yield further insights into the metabolism of homocysteine. For example, it is interesting to note that there is an observational association between homocysteine and clinical outcomes of a recent novel disease—Sars-Cov2 infection (108). Evidence relating one-carbon metabolism to coronavirus replication is in its infancy, but such replication requires single carbon units; the viral “machinery” has evolved its own methyltransferase to scavenge the host’s methyl units (109). It is likely that coming decades will reveal additional insights in relation to homocysteine and viral replication.

To close, we would point out that medical knowledge is expanding exponentially. Whereas the “doubling time” was estimated to be 50 years in the 1950’s, it is reported to have accelerated to 7 years by 1980, and 3.5 years by 2010 (110). Whatever the future brings, we can anticipate many exciting developments regarding homocysteine research over the next 90 years.

## Author contributions

All authors listed have made a substantial, direct, and intellectual contribution to the work and approved it for publication.

## Funding

Article Publication Costs were covered by COBALZ Limited, Wrexham, United Kingdom.

## Conflict of interest

AM is a shareholder and Scientific Advisor for COBALZ Limited, a private Limited Company developing novel B-vitamin and antioxidant supplements. JM receives compensation as Associate Editor for the journal Nutrition Reviews, and has received within the last 3 years consulting compensation from Church and Dwight, Inc., a producer and seller of consumer goods including vitamin supplements.

The study received funding from COBALZ Limited. The funder had the following involvement: APC costs.

## Publisher’s note

All claims expressed in this article are solely those of the authors and do not necessarily represent those of their affiliated organizations, or those of the publisher, the editors and the reviewers. Any product that may be evaluated in this article, or claim that may be made by its manufacturer, is not guaranteed or endorsed by the publisher.
